# PERPETRATOR DEMOGRAPHICS ASSOCIATED WITH ADULT PROTECTIVE SERVICES—CONFIRMED FINANCIAL EXPLOITATION CASES

**DOI:** 10.1093/geroni/igz038.1781

**Published:** 2019-11-08

**Authors:** Cassandra J Enzler, Carlos Reyes-Ortiz, and Jason Burnett

**Affiliations:** University of Texas Health McGovern Medical School, Houston, Texas, United States

## Abstract

Financial exploitation (FE) in older adults is a significant public health problem linked to outcomes including depression, financial ruin and early mortality. Studies have demonstrated risk factors associated with FE, but less is known about perpetrator characteristics. We performed a secondary data analysis of over 16,000 reported cases of FE utilizing a cross sectional design. Using multivariate logistic regression, confirmed and unconfirmed cases of FE were predicted from the following perpetrator demographics: age, gender, marital status, ethnicity, relationship to the victim, living status, and histories of drug abuse, alcohol abuse, and mental illness. Significant perpetrator demographics predicting confirmed FE were separation/divorce (OR=1.48), identifying as White (OR=1.33) or Black (OR=1.44), being a daughter (OR=1.61), son (OR=1.75), grandchild (OR=2.72), or other family member (OR=1.41), not residing with the victim (OR=2.32), and having a history of drug abuse (OR=2.56), alcohol abuse (OR=1.80), or mental illness (OR=1.91). These findings are based on a large statewide dataset and describe important perpetrator characteristics that could potentially be targeted for both intervention and prevention programs. This is especially important as many victims are reluctant to seek criminal action against a family member or trusted individual. This information is valuable as it may help APS, who has limited funding and staff, investigate and intervene in more difficult elder abuse FE cases.

